# Exogenous Alpha-Synuclein Evoked Parkin Downregulation Promotes Mitochondrial Dysfunction in Neuronal Cells. Implications for Parkinson’s Disease Pathology

**DOI:** 10.3389/fnagi.2021.591475

**Published:** 2021-02-24

**Authors:** Anna Wilkaniec, Anna M. Lenkiewicz, Lidia Babiec, Emilia Murawska, Henryk M. Jęśko, Magdalena Cieślik, Carsten Culmsee, Agata Adamczyk

**Affiliations:** ^1^Department of Cellular Signalling, Mossakowski Medical Research Centre (PAN), Polish Academy of Sciences, Warsaw, Poland; ^2^Institute of Pharmacology and Clinical Pharmacy, Philipps-University of Marburg, Marburg, Germany

**Keywords:** α-synuclein (α-syn), parkin, mitochondria dysfunction, mitophagy, PGC-1 alpha, Parkinson’s disease

## Abstract

Aberrant secretion and accumulation of α-synuclein (α-Syn) as well as the loss of parkin function are associated with the pathogenesis of Parkinson’s disease (PD). Our previous study suggested a functional interaction between those two proteins, showing that the extracellular α-Syn evoked post-translational modifications of parkin, leading to its autoubiquitination and degradation. While parkin plays an important role in mitochondrial biogenesis and turnover, including mitochondrial fission/fusion as well as mitophagy, the involvement of parkin deregulation in α-Syn-induced mitochondrial damage is largely unknown. In the present study, we demonstrated that treatment with exogenous α-Syn triggers mitochondrial dysfunction, reflected by the depolarization of the mitochondrial membrane, elevated synthesis of the mitochondrial superoxide anion, and a decrease in cellular ATP level. At the same time, we observed a protective effect of parkin overexpression on α-Syn-induced mitochondrial dysfunction. α-Syn-dependent disturbances of mitophagy were also shown to be directly related to reduced parkin levels in mitochondria and decreased ubiquitination of mitochondrial proteins. Also, α-Syn impaired mitochondrial biosynthesis due to the parkin-dependent reduction of PGC-1α protein levels. Finally, loss of parkin function as a result of α-Syn treatment induced an overall breakdown of mitochondrial homeostasis that led to the accumulation of abnormal mitochondria. These findings may thus provide the first compelling evidence for the direct association of α-Syn-mediated parkin depletion to impaired mitochondrial function in PD. We suggest that improvement of parkin function may serve as a novel therapeutic strategy to prevent mitochondrial impairment and neurodegeneration in PD (thereby slowing the progression of the disease).

## Introduction

Parkinson’s disease (PD) is a widespread progressive movement disorder and one of the most common neurodegenerative diseases, characterized by a degeneration of dopaminergic neurons in the substantia nigra pars compacta (SNpc) projecting into the basal ganglia. The key pathological features underlying the pathology of this disease are the deposition of α-synuclein (α-Syn) in Lewy bodies (LB) and mitochondria dysfunction. Over the past years, a variety of pathogenic mechanisms of PD have been proposed, including oxidative stress and disruption of calcium homeostasis, as well as proteolytic stress related to the impairment of the ubiquitin-proteasome system (UPS; Pringsheim et al., 2014; Jesko et al., 2019).

One of the important factors involved in the etiology of PD is the misfolding of soluble monomeric α-Syn into insoluble fibrils as well as a variety of post-translational modifications. Moreover, point- and copy-number mutations in the SNCA gene that encodes α-Syn have now been linked to autosomal dominant PD (Polymeropoulos et al., 1997; Krüger et al., 1998; Singleton et al., 2003; Chartier-Harlin et al., 2004; Zarranz et al., 2004; Appel-Cresswell et al., 2013; Lesage et al., 2013). A plethora of *in vitro* and *in vivo* studies have demonstrated that α-Syn may self-propagate between cells in a prion-like manner and proposed possible mechanisms of cell-to-cell transmission of this protein (Desplats et al., 2009; Prusiner et al., 2015). The excessive release of α-Syn into the extracellular space, driven by environmental factors as well as neural demise/neuronal disintegration, may have a significant role in the spread of neurodegeneration in the brain (Wilkaniec et al., 2013).

The E3 ubiquitin ligase parkin is another PD-associated protein that plays an important role in the ubiquitin-proteasome system and acts as a regulator of protein breakdown. Upon mitochondrial membrane depolarization, parkin is translocated to the mitochondrial surface, where it mediates the degradation of defective mitochondria in a process of regulated mitophagy (Narendra et al., 2008; Hammerling et al., 2017). Therefore, parkin is essential for mitochondrial quality control, integrity, and turnover. Parkin mutation or functional inactivation leads to the accumulation of misfolded, aggregated proteins and damaged mitochondria (Kitada et al., 1998; Chung et al., 2004; Yao et al., 2004; Wang et al., 2005; Wong et al., 2007).

To date, only a few studies have investigated the direct association between α-Syn and parkin dysfunctions, although mutations in both proteins have long been considered major causes of hereditary PD. Recently, we reported that extracellular α-Syn oligomers induce deregulation of parkin activity through S-nitrosylation with the subsequent degradation of this protein (Wilkaniec et al., 2019), thus providing evidence for a close connection between parkin dysfunction and extracellular α-Syn signaling in PD pathophysiology. While many studies have highlighted the direct association of the pathological pool of α-Syn with mitochondrial dysfunction (Banerjee et al., 2010; Wilkaniec et al., 2013; Ganjam et al., 2019), it is largely unknown whether impairment in parkin-dependent mitophagy might have an important role in PD, especially since it has been demonstrated that parkin knockout mice did not display a neurodegenerative phenotype (Pickrell and Youle, 2015). However, in a mouse model with accelerated mtDNA mutations resulting in the accumulation of dysfunctional mitochondria, the absence of parkin caused a dramatic loss of DA neurons in the SNpc (Pickrell et al., 2015). Thus, the requirement for severe mitochondrial stress in addition to the loss of parkin function to observe the neurodegenerative phenotype leaves open a question about the etiology of idiopathic PD in humans. Therefore, this study was designed to study whether exogenous α-Syn might be the trigger that leads to parkin-related mitochondrial damage.

## Materials and Methods

### Preparations of Oligomers and Protofibrils

Human recombinant lyophilized α-synuclein (α-Syn) was obtained from rPeptide (Bogart, GA, USA). α-Syn oligomers were prepared according to Danzer et al. (2007) with modifications. Briefly, α-Syn was dissolved to a 7 μM concentration in 50 mM sodium phosphate buffer (PB), pH 7.0, containing 20% ethanol. After 4 h of shaking (1,000 rpm; room temperature, RT), oligomers were re-lyophilized and resuspended with one-half the starting volume of 50 mM PB, pH 7.0, containing 10% ethanol. This was followed by stirring (with open lids to evaporate residual ethanol) for 24 h at RT under a sterile hood. The concentration of obtained α-Syn oligomers was then measured using a NanoDrop 2000 spectrophotometer (Thermo Fisher Scientific).

### Cell Culture and Differentiation

The studies were carried out using rat pheochromocytoma (PC12) cells. PC12 cells were cultured in Dulbecco’s Modified Eagle’s Medium (DMEM) supplemented with 10% heat-inactivated FBS, 5% heat-inactivated HS, 50 units/ml penicillin, and 50 μg/ml streptomycin and L-glutamine at 37°C in a humidified incubator in a 5% CO_2_ atmosphere. For neuronal differentiation, PC12 cells were treated with 50 ng/ml NGF in a low-serum medium (DMEM supplemented with 2% FBS, 1% penicillin/streptomycin, and 1% L-glutamine) every 24 h for 96 h. Every 48 h, culture media was removed and replaced with fresh media and NGF according to Binnington and Kalisch (2007).

### Stable, Constitutive Overexpression of Parkin in PC12 Cells

The sequence encoding human wild-type parkin was subcloned into AscI/PacI sites of the pcDNA4.3Asc vector. The construct was evaluated first using AscI/PacI restriction analysis, and then with a western blot on extracts of transiently transfected CHO cells with antibodies against parkin (Santa Cruz, SC-32282). PC12 cells were electroporated at 5 × 10^6^ per cuvette (BioRad Gene PulserXcell) with 10 μg of empty-vector (pcDNA) or pcDNA vector with parkin sequence (pcDNA-Parkin), using VWR cuvettes with a 4 mm gap and one 30 ms pulse of 220 V. The pcDNA and pcDNA-parkin cells were plated in a culture medium and propagated with one medium change until reaching 90% confluency. Then, the selection was started using G418 (30 μg/ml initial concentration, gradually increased to 100 μg/ml). After the cells accumulated, the clonal selection was performed as previously described (Wilkaniec et al., 2019).

### siRNA Mediated Parkin Knock-Down

For RNA interference, NGF-treated PC12 cells were transfected with appropriate siRNA: PARK-2 (L-090709-02; Dharmacon) or control (D-001810-10-05; Dharmacon) using Lipofectamine RNAiMAX (Invitrogen) according to the manufacturer’s protocol. The expression of parkin in transfected cells was then examined by western blot.

### Cellular Treatment

PC12 cells were seeded into 100-mm, 60-mm, and 35-mm culture dishes, 24-well or 96-well plates coated with 0.1% PEI or rat tail collagen, and the growth medium was changed to a low-serum medium (DMEM supplemented with 2% FBS, 1% penicillin/streptomycin and 1% L-glutamine). HBSS or other media appropriate for the particular procedure were also used. Then, the cells were treated with exogenous α-Syn oligomers (5 μM) for appropriate time points. A suitable solvent was added to appropriate controls.

### Isolation of Mitochondrially Enriched and Cytosolic Fractions

Cells were seeded in a 10 cm culture dish at a density of 5 × 10^6^. After 24 h of incubation in the presence of tested compounds, cells were washed with ice-cold PBS and resuspended for 15 min on ice in a permeabilization buffer containing 75 mM NaCl, 1 mM NaH_2_PO_4_, 8 mM Na_2_HPO_4_, 250 mM sucrose, 1 mM PMSF, 0.05% Triton X-100, and Complete^®^ protease inhibitor mixture tablets (Roche Diagnostics). Then, the cells were homogenized with a glass homogenizer, and the resulting homogenate was centrifuged at 800× *g* for 10 min at 4°C to remove nuclei and tissue particles. The supernatant 1 (S1) was saved and the pellet was resuspended in the lysis buffer. The homogenization and low-speed centrifugation steps were repeated. The supernatant 2 (S2) was saved and added to supernatant 1. The combined mitochondria-enriched supernatants (S1 + S2) were centrifuged at 20,000× *g* for 15 min at 4°C to obtain the mitochondrial fraction. The supernatant 3 (S3) was used as a cytosolic fraction, and the pellet was resuspended in PBS. Both fractions were stored at −20°C until use, followed by determination of protein content by Bradford Reagent (Merck, Kenilworth, NJ, USA).

### Measurement of Mitochondrial ROS Production Using Mitosox Red

Mitochondrial superoxide production was measured using the MitoSOX Red fluorescent probe according to Kauffman et al. (2016), with modifications. Cells were plated in eight replicates into a black 96-well cell culture plate at a density of 1.5 × 10^4^ cells/well. After 24 h incubation in the presence of tested compounds, cells were washed twice with HBSS to remove the medium and subsequently incubated for 10 min (needed to allow the probe to enter the cell and start the reaction within the mitochondria) at 37°C in 100 μl of measurement buffer containing 2.5 μM MitoSOX Red. After incubation, the cells were washed twice with HBSS. The fluorescence was monitored in the measurement buffer with a Tecan Infinite M200 plate reader (Tecan US Inc., Durham, NC, USA) set to 510 nm excitation (Ex bandwidth: 10 nm) and 595 nm emission (Em bandwidth: 35 nm) wavelengths.

### Determination of Mitochondrial Membrane Potential

The mitochondrial membrane potential in PC12 cells was monitored using lipophilic probe JC-1 followed by flow cytometric detection. PC12 cells were plated at a density of 1 × 10^6^ cells per 6 cm dish. After 24 h incubation in the presence of tested compounds, cells were detached with Accutase^®^ and stained using a BD^TM^ MitoScreen (JC-1) kit according to the manufacturer’s protocol. JC-1 accumulates within intact mitochondria to form multimer J-aggregates (red color; *λ*ex = 488 nm, *λ*em = 590 nm), and the color of the dye changes from red to green (*λ*ex = 488 nm, *λ*em = 530 nm) due to depolarization of mitochondrial membrane potential. This alteration was analyzed on flow cytometer FACS Canto II using FACSDiva Software (BD Biosciences, San Jose, CA, USA). The ratio of aggregate (*λ*em = 590 nm) and monomer (*λ*em = 530 nm) fluorescence was used as a measure of mitochondrial depolarization (ΔΨm).

### Measurement of Mitochondrial Mass by Flow Cytometry

The measurement of mitochondrial mass was based on the selective accumulation of Mitotracker Green (MTG) dye in the mitochondrial matrix regardless of mitochondrial potential (Doherty and Perl, 2017). PC12 cells were plated at a density of 1 × 10^6^ cells per 6 cm dish. After 8, 12, or 24 h incubation in the presence of tested compounds, cells were detached with Accutase^®^ solution (Merck, Kenilworth, NJ, USA) and subsequently incubated for 45 min at 37°C in of HBSS containing 100 nM MitoTracker Green (Thermo Fisher Scientific, Waltham, MA, USA). After the incubation, the cells were washed twice with HBSS. The fluorescence (ex490, em516) was monitored in the measurement buffer with a FACS Canto II using FACSDiva Software (BD Biosciences, San Jose, CA, USA).

### Measurement of ATP Levels

The total ATP content of PC12 cells was determined using a bioluminescence assay (ViaLigh^TM^ Plus Kit, Lonza, Basel, Switzerland) according to the manufacturer’s instructions. The kit is based upon the bioluminescent measurement of ATP that is present in all metabolically active cells. The bioluminescent method utilizes an enzyme, luciferase, which catalyzes the formation of light from ATP and luciferin. PC12 cells were plated in 8 replicates into a white 96-well cell culture plate at a density of 1.5 × 10^4^ cells/well. After 24 h incubation in the presence of tested compounds, the cells were lysed for 10 min RT and the AMR plus reagent was added. After 2 min of incubation RT, the bioluminescence was measured using a fluorescence spectrophotometer (FLUOstar Omega; BMG LABTECH, Ortenberg, Germany).

### Determination of Mitochondrial Redox Environment

To investigate changes in the mitochondrial redox environment, PC12 cells were transfected with a plasmid coding for a redox-sensitive green fluorescent protein with a mitochondrial targeting sequence (pRA306 in pEGFP-N1). In an oxidized environment, absorption increases at short wavelengths (375 nm) at the expense of absorption at longer wavelengths (500 nm). The fluorescence ratio indicates oxidation/reduction as described previously by Hanson et al. (2004). PC12 cells were transfected using electroporation (Neon Transfection System) in 100 μl volume containing 1.4 × 10^6^ cells and 20 μg DNA, at the manufacturer’s PC12-optimized pulse parameters (Thermo Fisher Scientific). Cells were plated in four replicates onto 96-well plates at a density of 1.5 × 10^4^ cells/well in standard culture medium (less antibiotics) and kept overnight at 37°C in 5% CO_2_. After 24 h treatment with oligomeric α-Syn, cells were washed twice with PBS and placed in Hank’s buffer. The ratio 375 nm/500 nm was measured using the multiplate reader Infinite M1000 PRO (TECAN). An increase in the ratio indicates a more oxidized environment.

### Isolation of DNA, RNA, and Reverse Transcription

For mitochondrial and genomic DNA isolation, PC12 cells were plated at a density of 1 × 10^6^ cells per 6 cm dish. After 24 h incubation in the presence of tested compounds, the cells were collected and centrifuged (17,000× *g*, 5 min, RT). Total DNA was isolated from the cells under sterile conditions using the commercial Genomic Mini kit (A&A Biotechnology, Poland) according to the manufacturer’s instruction.

The total RNA isolation was performed according to the procedure developed by Chomczyński using TRI Reagent^®^ (cat. T9424) from Sigma–Aldrich, following the manufacturer’s protocol. Digestion of DNA contamination was performed using DNase I according to the manufacturer’s protocol (Sigma–Aldrich, St. Louis, MO, USA). RNA quantity and quality were controlled by spectrophotometric analysis and gel electrophoresis. A reverse transcription was performed by using the high-capacity cDNA reverse transcription kit according to the manufacturer’s protocol (Applied Biosystems, Foster City, CA, USA).

### Quantitative Real-Time Polymerase Chain Reaction (qRT-PCR)

Quantitative real-time PCR was performed with TaqMan Universal PCR Master Mix (Applied Biosystems, Foster City, CA, USA) and detected by a Real-Time PCR System on an ABI PRISM 7500 apparatus (Thermo Fisher Scientific, Waltham, MA, USA) using the commercially available TaqMan^®^ Gene Expression Assays (*Actb* Rn01412977_g1; *Mfn2* Rn00500120_m1; *Tfam Rn00580051_m1, Nrf-1* R*n01455958_m1, Gapdh Rn01775763g1, mt-Atp*6 R*n03296710_s1*). *Actb* or *Gapdh* were used in the analysis as reference genes. A standard two-step PCR amplification was performed, with a melting step at 95°C for 15 s and annealing and elongation at 60°C for 1 min, for 40 cycles. The relative levels of target mRNA or DNA, normalized to an endogenous reference and relative to a calibrator, were calculated by a 2^−ΔΔCT^ formula.

### Western Blot Analysis

The cells were washed twice with ice-cold PBS and lysed in cell lysis buffer (1×). Protein levels were determined using the Bradford method, and then the samples were mixed with Laemmli buffer and denatured at 95°C for 5 min. Equal amounts of proteins were separated on SDS/PAGE gels. All proteins were transferred to nitrocellulose membranes at 100V. Membranes were washed for 5 min in TBS-Tween buffer (0.1% TBST; 100 mM Tris-buffered saline, 140 mM NaCl, and 0.1% Tween 20; pH 7.6) and the nonspecific bindings were blocked for 1 h at RT with 5% BSA in 0.1% TBST or with 5% non-fat milk solution in 0.1% TBST. Immunodetection was performed overnight at 4°C using rabbit anti-Drp1 (1:1,000, sc-32898, Santa Cruz), rabbit anti-Mfn1 (1:500, Ab104585, Abcam), rabbit anti-Mfn2 (1:1,000, sc-50331, Santa Cruz), rabbit anti-Opa-1 (1:1,000, sc-367890, Santa Cruz), rabbit anti-parkin (1:500; #2132S, Cell Signaling), rabbit anti-PGC-1α (1:1,000, sc-13067, Santa Cruz), rabbit anti-ubiquitin (1:500, 07-375, Merck Millipore), rabbit anti-p62 (1:500; #5114, Cell Signaling) and anti-LC3-I/II (1:1,000, L8918, Sigma–Aldrich, St. Louis, MO, USA) antibodies. Then, the membranes were washed three times (5 min) in TBST and incubated for 60 min at RT with anti-rabbit secondary antibody (1:4,000, #7074P2, Cell Signaling) in a 5% non-fat milk/TBST. Antibodies were detected using chemiluminescent Clarity Western ECL Substrate (Bio-Rad Laboratories, Hercules, CA, USA) under standard conditions. Immunolabeling of GAPDH (rabbit anti-GAPDH; 1:40,000; G9545, Sigma–Aldrich) or VDAC (rabbit anti-VDAC; 1:1,000, AB10527 Merck Millipore) for cell lysates and Ponceau-S staining for the mitochondrial fraction was performed as a loading control.

### Monitoring of Mitochondrial Autophagy (Mitophagy)

For mitophagy, PC12 cells were plated in four replicates into a glass-bottomed, four-component, 35 mm CellView^®^ culture dish at a density of 1 × 10^5^ cells. After 24 h incubation in the presence of tested compounds, the cells were stained with MitoTracker Green (100 nM, Thermo Fisher Scientific) and LysoTracker Red (100 nM, Thermo Fisher Scientific) and imaged with inverted LSM 510 or Axio Observer Z.1 confocal microscopes (Zeiss) at 63× magnification using the linear sequential scan mode function (excitation/emission filter, 488/510 nm; 543/592 nm). Colocalization analyses (Pearson’s correlation coefficient) were performed using the JACoP plugin in ImageJ (Bolte and Cordelières, 2006).

### Quantification of Mitochondrial Morphology

PC12 cells transfected with pRA306 were plated in four replicates into a glass-bottomed, four-component, 35 mm CellView^®^ culture dish at a density of 1 × 10^5^ cells. After 24 h incubation in the presence of tested compounds, live cells were imaged with an inverted LSM 510 confocal microscope (Zeiss) using 488 nm Argon laser excitation, and RGB images were captured at a magnification of 63×. The publically available ImageJ macro created by Dagda et al. (2009) was used to quantify two parameters of mitochondrial morphology. Briefly, the green channel of PC12 cells expressing pRA306 was extracted to grayscale, inverted to show mitochondria-specific fluorescence as black pixels, and thresholded to optimally resolve individual mitochondria. The macro traces mitochondrial outlines using “analyze particles.” The form factor (perimeter^2^/[4π × area]) and the aspect ratio (ratio between the major and minor axes of the ellipse equivalent to the object) were calculated representing mitochondrial interconnectivity and elongation.

### Statistical Analysis

The results were expressed as mean values ± SD. Differences between the means were analyzed using a Student’s *t*-test between two groups and two-way analysis of variance (ANOVA) with Bonferroni comparison *post hoc* test among multiple groups. Statistical significance was accepted at *p* < 0.05. The statistical analyses were performed using Graph Pad Prism version 5.0 (Graph Pad Software, San Diego, CA, USA).

## Results

### α-Syn Impairs Mitochondrial Bioenergetics Through Down-Regulation of Parkin

**Figure 1:**
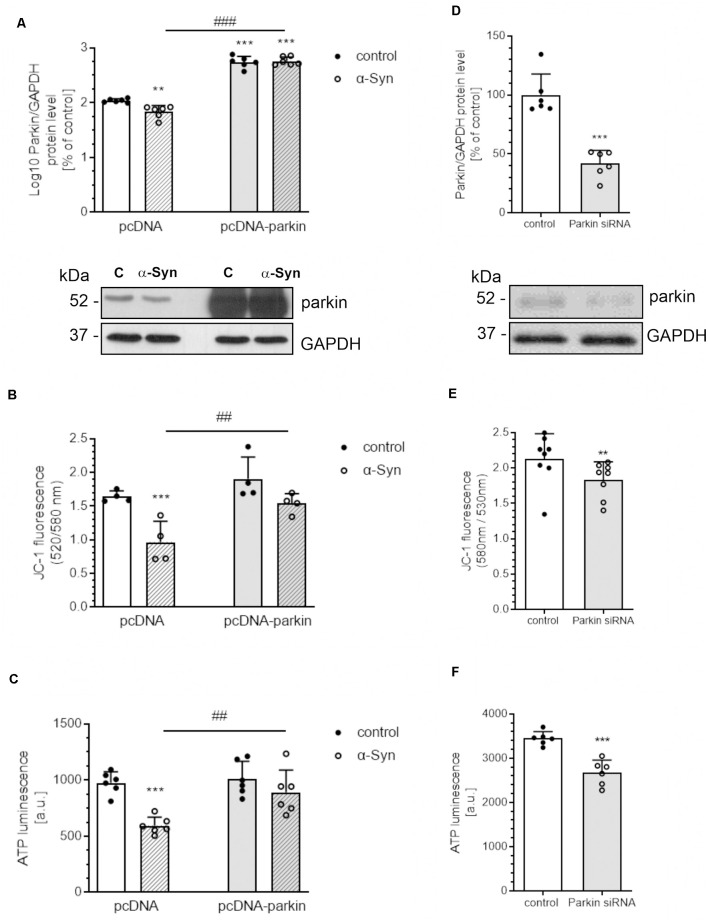
α-Syn-mediated parkin depletion is responsible for mitochondrial dysfunction in PC12 cells. **(A)** Parkin immunoreactivity normalized to GAPDH in pcDNA and pcDNA-Parkin PC12 cells treated with α-synuclein (α-Syn) for 24 h at a concentration of 5 μM. Data were log10 transformed and represent the mean value ± SD for 6 independent experiments (*n* = 6). **p < 0.01, ***p < 0.001 compared to pcDNA control cells; ^###^*p* < 0.001 compared to pcDNA cells treated with α-Syn, using two-way ANOVA followed by Bonferroni *post hoc* test. **(B)** Mitochondrial membrane potential (ΔΨm) and **(C)** ATP levels were measured after α-Syn oligomers treatment for 24 h at a concentration of 5 μM in pcDNA and pcDNA-Parkin PC12 cells. Data represent the mean value ± SD (B—*n* = 4, C—*n* = 6). ***p < 0.001 compared to pcDNA control cells; ^##^*p* < 0.01, compared to pcDNA cells treated with α-Syn, using two-way ANOVA followed by Bonferroni *post hoc* test. **(D)** Parkin immunoreactivity normalized to GAPDH in Parkin knock-down PC12 cells. Data were normalized to the corresponding untreated control group (=100%) and represent the mean value ± SEM for six independent experiments (*n* = 6). ***p < 0.001 compared to corresponding control siRNA, using Student’s *t*-test. **(E)** Mitochondrial membrane potential (ΔΨm) and **(F)** ATP levels were measured in Parkin knock-down PC12 cells. Data represent the mean value ± SD (E—*n* = 8, F—*n* = 6). **p < 0.01, ***p < 0.001 compared to corresponding control siRNA, using Student’s *t*-test.

In view of the previous report showing the negative impact of extracellular α-Syn on parkin protein level in dopaminergic cells (Wilkaniec et al., 2019), we tested the hypothesis as to whether this deregulation of parkin significantly contributes to mitochondrial dysfunction. The experiments were conducted on the rat pheochromocytoma (PC12) cell line, which can synthesize, store, and release catecholamines, such as CNS dopaminergic neurons (Greene and Tischler, 1976). Moreover, upon treatment with nerve growth factor (NGF), those cells undergo neuronal differentiation, which results in a series of phenotypic changes characteristic of sympathetic neurons (Malagelada and Greene, 2008). To examine the role of parkin in alterations of mitochondrial bioenergetics induced by *in vitro*-generated oligomeric α-Syn species, we used PC12 cells transfected with the human parkin gene (pcDNA-Parkin), which synthesize about four times more parkin (422.9 ± 45.09) than control cells transfected with the empty vector (pcDNA: 100 ± 7.3; Figure 1A). Due to large discrepancies in parkin immunoreactivity between the pcDNA and pcDNA-Parkin cells, the obtained data were log10 transformed for statistical purposes. Both of the investigated cell lines were differentiated with NGF for 96 h, to observe the neuronal phenotype (Supplementary Figure 1). We observed that, upon an α-Syn treatment, a significant decrease in parkin immunoreactivity in pcDNA control cells occurs, whereas in pcDNA-Parkin PC12 cells α-Syn treatment does not have a substantial effect on parkin protein level (Figure 1A). To establish the involvement of parkin in functional impairment of mitochondria, the mitochondrial membrane potential (MMP; the determinant of mitochondria polarization state) and ATP cellular levels (the indicator of oxidative phosphorylation) were measured in PC12 cells. We observed that a 24-h treatment of control pcDNA cells with exogenous α-Syn oligomers results in a substantial loss of MMP (Figure 1B), together with a significant decrease in cellular ATP level (Figure 1C), whereas parkin overexpression prevents α-Syn-evoked depolarization of mitochondrial membrane potential and a decline in ATP level (Figures 1B,C). To determine whether the depletion of parkin levels may be a direct cause of α-Syn-induced imbalances in mitochondrial homeostasis, we silenced endogenous parkin with siRNA in naïve PC12 cells (siRNA-Parkin). Parkin levels in transiently transfected cells were reduced by ~60% (Figure 1D). In parkin-depleted cells, mitochondrial membrane potential (Figure 1E) and ATP levels (Figure 1F) were significantly decreased, but this effect was less pronounced when compared to PC12 cells treated with α-Syn (Figures 1B,C).

**Figure 2:**
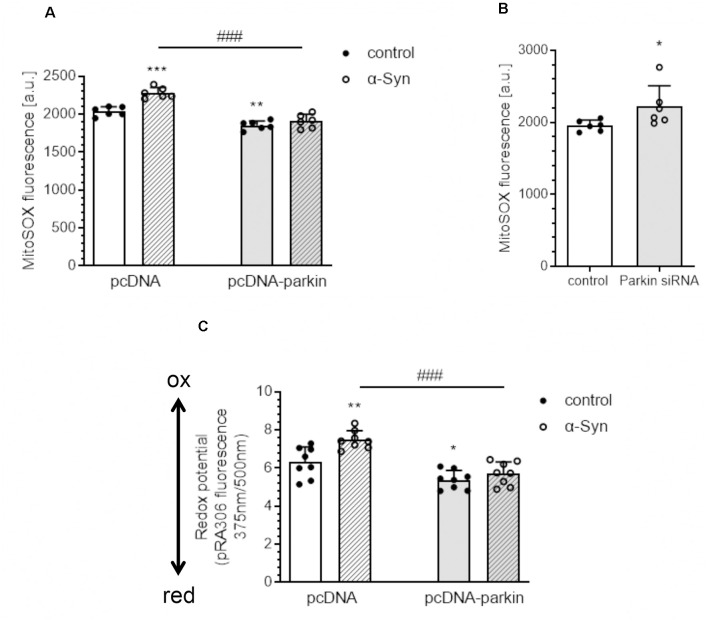
Parkin deregulation causes the disruption of the homeostasis of mitochondrial redox state in PC12 cells after α-Syn treatment. **(A)** Mitochondrial reactive oxygen species (mtROS) levels were measured after α-Syn oligomers treatment for 24 h at a concentration of 5 μM in pcDNA and pcDNA-Parkin PC12 cells. Data represent the mean value ± SD for six independent experiments (*n* = 6). **p < 0.01, ***p < 0.001 compared to pcDNA control cells; ^###^*p* < 0.001 compared to pcDNA cells treated with α-Syn, using two-way ANOVA followed by Bonferroni *post hoc* test. **(B)** Mitochondrial reactive oxygen species (mtROS) levels were measured in Parkin knock-down PC12 cells. Data represent the mean value ± SD for six independent experiments (*n* = 6). *p < 0.05, compared to corresponding control siRNA, using Student’s *t*-test. **(C)** Using PC12 cells overexpressing the redox-sensitive green fluorescent protein (pRA306 roGFP) located within mitochondria, the mitochondrial redox state was measured after α-Syn oligomers treatment for 24 h at a concentration of 5 μM in pcDNA and pcDNA-Parkin PC12 cells. Data represent the mean value ± SD for eight independent experiments (*n* = 8). *p < 0.05, **p < 0.01 compared to pcDNA control cells; ^###^*p* < 0.001 compared to pcDNA cells treated with α-Syn, using two-way ANOVA followed by Bonferroni *post hoc* test.

Subsequently, we evaluated the mitochondrial reactive oxygen species (mtROS) level and measured the mitochondrial redox state as an indicator of oxidative stress in PC12 cells treated with exogenous α-Syn. Using a fluorogenic dye MitoSOX^TM^ Red, we observed that exogenous α-Syn induces a significant increase in the mitochondrial superoxide level in pcDNA cells, whereas, in parkin overexpressing cells, the levels of mtROS were markedly reduced, either in basal conditions or after α-Syn treatment (Figure 2A). Moreover, the superoxide anion levels were also augmented upon parkin silencing (Figure 2B). In the following studies, by using PC12 cells transfected with a redox-sensitive green fluorescent protein-harboring mitochondrial targeting sequence (pRA306 GFP), we evaluated whether parkin downregulation is involved in the changes of the mitochondrial redox state evoked by α-Syn oligomers (Figure 2C). Together with an increase of mtROS, we detected significant elevation of oxidative stress in mitochondria 24 h after α-Syn treatment in pcDNA cells, whereas parkin overexpression counteracted the deregulation of the mitochondrial redox state induced by α-Syn oligomers (Figure 2C).

Together, these data show that, upon an exogenous α-Syn treatment, various functional defects of mitochondrial activity and deregulation of mitochondrial redox homeostasis in neuronal cells are directly associated with a decrease in parkin level.

### Role of Parkin in Mitochondrial Network Deregulation by α-Syn

**Figure 3:**
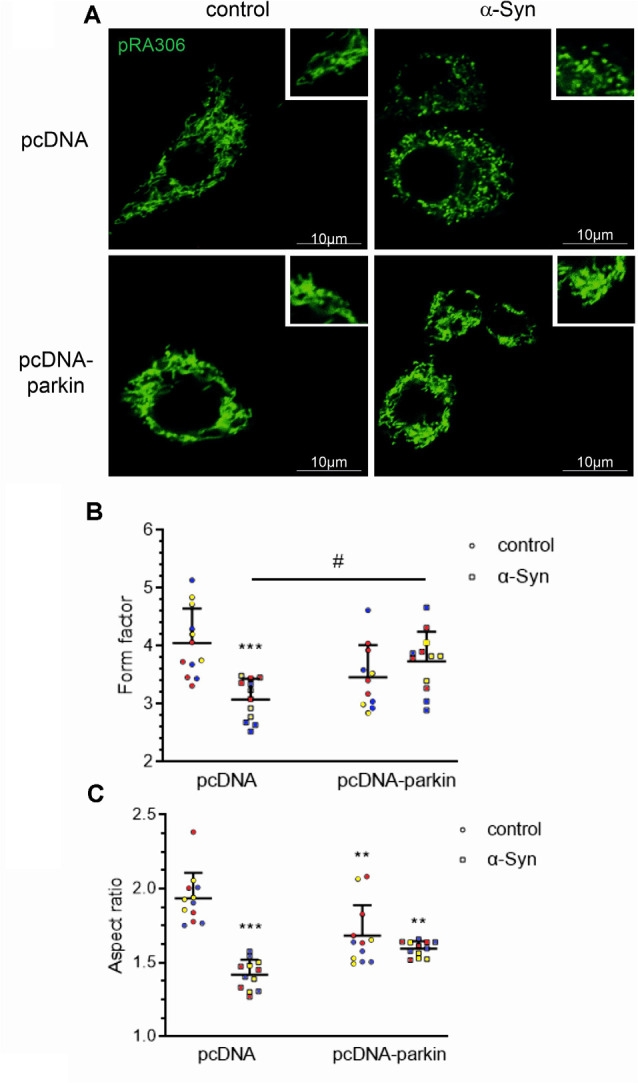
Parkin deregulation is involved in the alteration of mitochondrial morphology in PC12 cells treated with α-Syn. **(A)** Epifluorescence images of mitochondria labeled by transient transfection of pRA306 roGFP in stable pcDNA and pcDNA-Parkin PC12 cells treated with α-Syn for 24 h at a concentration of 5 μM. Scale bar: 10 μm. Quantitative image analysis of **(B)** mitochondrial branching (form factor) and **(C)** length (aspect ratio) of pcDNA and pcDNA-Parkin PC12 cells treated with α-Syn. Data represent the mean value ± SD for a representative of three experiments with four fields per experiment (*n* = 12, different colors represent independent experiments). **p < 0.01, ***p < 0.001 compared to pcDNA control cells; ^#^*p* < 0.05 compared to pcDNA cells treated with α-Syn, using two-way ANOVA followed by Bonferroni *post hoc* test.

Mitochondria are highly mobile, dynamic organelles that undergo a continuous cycle of fusion-fission in response to cellular energy demands; the processes of mitochondrial network formation are critical components of the cellular stress response (Meyer et al., 2017). To investigate whether parkin deregulation evoked by exogenous α-Syn treatment interfered with mitochondrial dynamics, we assessed mitochondrial morphology in pRA306 overexpressing PC12 cells by confocal microscopy (Figure 3A). We observed that α-Syn treatment induced significant mitochondrial fragmentation, compared with control pcDNA cells, which showed a clear profile of tubular, interconnected mitochondria. Interestingly, in pcDNA-Parkin cells, we observed the formation of an “intermediate” shape of the mitochondrial network expressed through the presence of mixed tubular and spherical mitochondria. We observed a similar structure of mitochondria in pcDNA-Parkin cells treated with α-Syn (Figure 3A). Next, by using ImageJ software we quantified mitochondrial shape descriptors; form factor (ratio between their area and perimeter; Figure 3B), and aspect ratio (ratio between the major and minor axes of the analyzed particles; Figure 3C) to estimate the degree of branching and the length of mitochondria. We observed significantly shorter and less branched mitochondria in pcDNA cells treated with α-Syn, whereas the stable overexpression of parkin significantly restored mitochondrial branching, although it had a negligible effect on mitochondrial length in α-Syn-treated cells (Figures 3B,C).

**Figure 4:**
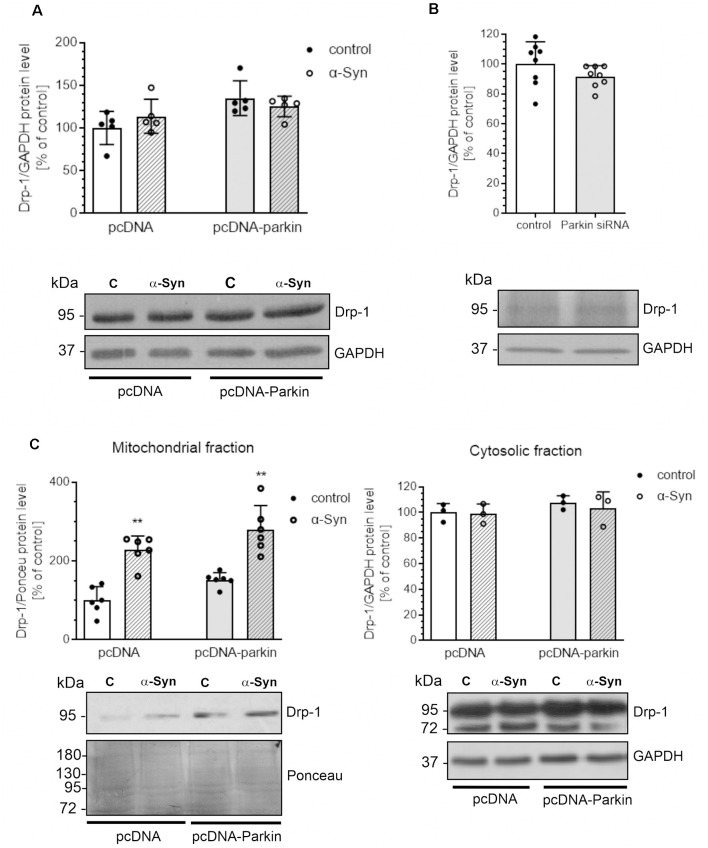
α-Syn treatment or parkin modifications do not change the protein level of Drp-1. Drp-1 immunoreactivity normalized to GAPDH in **(A)** pcDNA and pcDNA-Parkin PC12 cells treated with α-Syn for 24 h at a concentration of 5 μM, or in **(B)** Parkin knock-down PC12 cells. Data were normalized to the untreated control group (=100%) and represent the mean value ± SD (A—*n* = 5, B—*n* = 8). **(C)** The immunoreactivity of Drp1 protein in mitochondrial (normalized relative to Ponceau-S) and cytosolic (normalized to GAPDH) extracts from pcDNA and pcDNA-Parkin PC12 cells treated with α-Syn for 24 h at a concentration of 5 μM. Data represent the mean value ± SD (mitochondria—*n* = 6, cytosol—*n* = 3). **p < 0.01, compared to pcDNA control cells using two-way ANOVA followed by Bonferroni *post hoc* test.

Several proteins that regulate the morphology of the mitochondrial network have been identified, among which Mitofusin 1 (Mfn1) and Mitofusin 2 (Mfn2), and Optic atrophy 1 (Opa1) were shown to mediate mitochondrial fusion, the process responsible for restoring functional proteins and undamaged mitochondrial DNA to defective organelles (Meeusen et al., 2004; Song et al., 2009). Counterbalancing fusion is mitochondrial fragmentation, which is driven by translocation of Drp1 that forms a structure surrounding the mitochondrion, acting as a tightening ring in the mitochondrial membrane (Burté et al., 2015). Mitochondrial fission has significant implications for stress response and apoptosis (Chan, 2012) and constitutes the initial step of mitophagy (Itoh et al., 2013). To analyze whether the mitochondrial fragmentation evoked by α-Syn depends on mitochondrial fission machinery, we examined the level of Drp1 and observed that neither α-Syn treatment nor parkin overexpression significantly changed the total level of this protein (Figure 4A). Similarly, siRNA-mediated reduction in parkin level had no significant effect on the immunoreactivity of Drp1 (Figure 4B). However, we observed that treatment of pcDNA and pcDNA-Parkin cells with α-Syn resulted in enhanced Drp1 translocation to mitochondria (Figure 4C).

**Figure 5:**
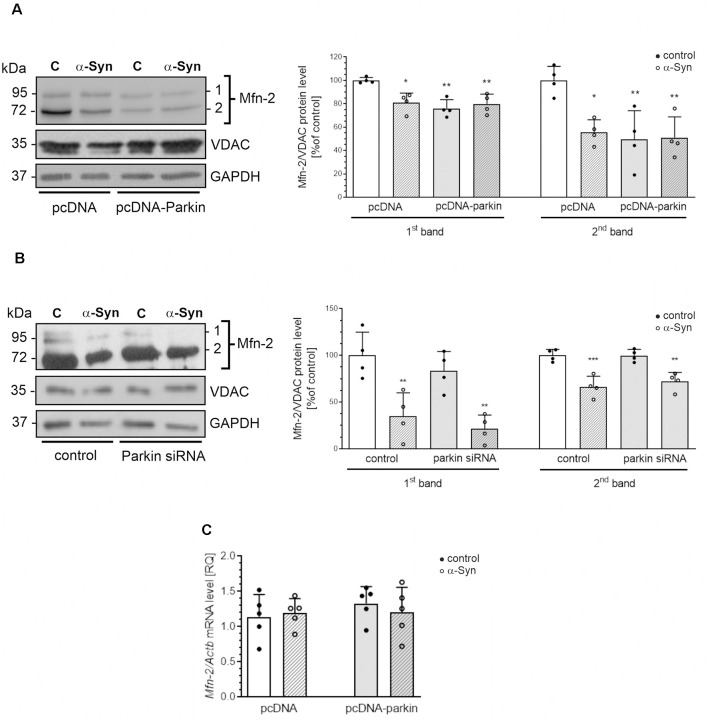
The effect of α-Syn treatment or parkin modifications on the protein expression of mitofusin-2. **(A)** The immunoreactivity of Mfn-2 normalized to VDAC (GAPDH was presented as a loading control) in pcDNA and pcDNA-Parkin PC12 cells treated with α-Syn for 24 h at a concentration of 5 μM. Data were normalized to the untreated control group (=100%) and represent the mean value ± SD for four independent experiments (*n* = 4). *p < 0.05, **p < 0.01, compared to pcDNA control cells using two-way ANOVA followed by Bonferroni *post hoc* test. **(B)** The immunoreactivity of Mfn-2 normalized to VDAC (GAPDH was presented as a loading control) in Parkin knock-down PC12 cells treated with α-Syn for 24 h at a concentration of 5 μM. Data were normalized to the untreated control group (= 100%) and represent the mean value ± SD for four independent experiments (*n* = 4). **p < 0.01, ***p < 0.001 compared to control cells using two-way ANOVA followed by Bonferroni *post hoc* test. **(C)** The mRNA level of Mfn-2 in pcDNA and pcDNA-Parkin PC12 cells treated with α-Syn for 24 h at a concentration of 5 μM. The mRNA level was measured by real-time PCR and normalized to Actb (β-actin). Data represent the mean value ± SD for five independent experiments (*n* = 5).

Additionally, in our experimental paradigm, we investigated changes in the levels of Opa1, as well as Mfn1 and Mfn2. We found that neither exogenous α-Syn treatment, parkin overexpression, nor silencing has a substantial impact on the immunoreactivity of Opa1 and Mfn1 (Supplementary Figure 2). Interestingly, we observed that both treatment of pcDNA cells with exogenous α-Syn and parkin overexpression significantly decrease the level of the two splicing forms (80 kDa and 60 kDa) of Mfn2. Moreover, treatment with exogenous α-Syn did not substantially change the effect of parkin overexpression on Mfn2 protein level (Figure 5A). We also observed that parkin down-regulation in PC12 cells did not affect the level of this protein (Figure 5B). Notably, in PC12 cells with parkin silencing, the inhibitory effect of exogenous α-Syn was similar to that obtained for control cells (Figure 5B). Altogether, those data suggest that exogenous α-Syn and parkin overexpression significantly reduced the level of Mfn2 in PC12 cells, and the inhibitory effect of α-Syn is rather independent of the decrease in parkin protein level. Finally, to test whether the changes in Mfn2 level resulted from the changes in gene expression, we measured Mfn2 mRNA levels in pcDNA and pcDNA-Parkin cells and observed that neither α-Syn treatment nor parkin overexpression had a significant impact on the total mRNA level of Mfn2 (Figure 5C).

### The Role of Parkin in α-Syn-Induced Deregulation of Mitophagy and Mitochondrial Biogenesis

**Figure 6:**
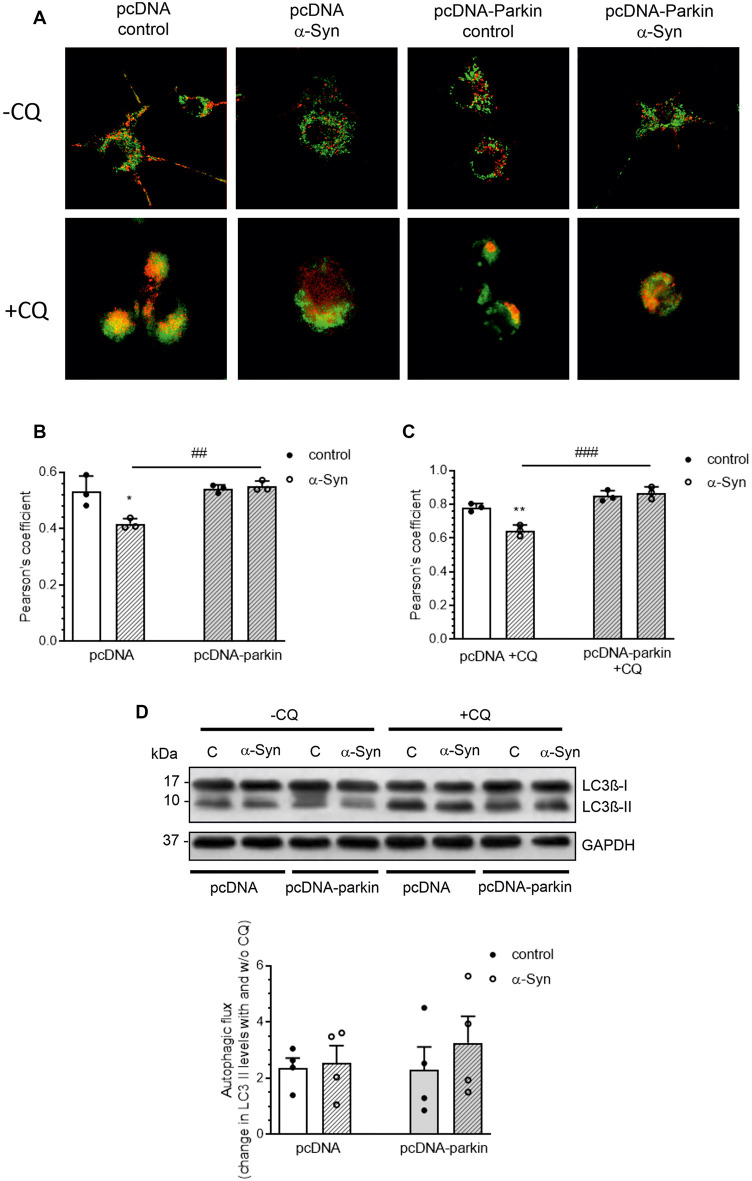
Parkin deregulation is involved in the alteration of mitochondrial autophagy in PC12 cells treated with α-Syn. **(A)** Single confocal captions showing an overlay of Lysotracker Red (LTR; red) and Mitotracker Green (MTG; green) in pcDNA and pcDNA-Parkin PC12 treated for 24 h with α-Syn at a concentration of 5 μM in the absence (top panel)/presence (bottom panel) of 40 μM chloroquine (CQ). Scale bar: 10 μm. The presented cells are representative of most of the analyzed cells. **(B)** Pearson’s colocalization coefficient (PCC) of LTR and MTG in PC12 cells treated for 24 h with α-Syn. Data were derived from three independent experiments with eight fields per experiment (*n* = 3). Each value is expressed as PCC ± SD *p < 0.05 compared to pcDNA control cells; ^##^*p* < 0.01 compared to pcDNA cells treated with α-Syn, using two-way ANOVA followed by Bonferroni *post hoc* test. **(C)** Pearson’s colocalization coefficient (PCC) of LTR and MTG in PC12 cells treated for 24 h with α-Syn in the presence of 40 μM CQ. Data were derived from three independent experiments with eight fields per experiment (*n* = 3). Each value is expressed as PCC ± SD **p < 0.01 compared to pcDNA control cells; ^###^*p* < 0.001 compared to pcDNA cells treated with α-Syn, using two-way ANOVA followed by Bonferroni *post hoc* test. **(D)** Immunoblotting of LC3-I and LC3-II in pcDNA and pcDNA-Parkin PC12 cells treated for 24 h with α-Syn at a concentration of 5 μM in the absence (left panel)/ presence (right panel) of 40 μM CQ for the last 2 h. Densitometric quantitation shows autophagic flux represented by a change in chloroquine-induced LC3-II levels. Data represent the mean value ± SD for four independent experiments (*n* = 4).

Growing evidence indicates that mitophagy, an autophagic clearance of damaged mitochondria, plays an important protective role in resistance to mitochondrial dysfunction-induced injury in disease states (Ding and Yin, 2012). As parkin has previously been found to play a pivotal role in this quality control mechanism, in the following studies, we investigated whether the classic mitophagy pathway in α-Syn-treated PC12 cells was functional. The number of mitochondria entrapped in autophagic vacuoles was visualized using a Mitotracker Green (MTG)/Lysotracker Red (LTR) staining technique. Colocalization of MTG and LTR was observed as yellow-orange puncta (Figure 6A), and it was quantified by the Pearson’s correlation coefficient (PCC; Figure 6B), the statistical tool that measures pixel-by-pixel covariance in a manner independent of signal levels and signal offset (background). Confocal images showed that α-Syn treatment of pcDNA cells markedly reduced the colocalization of the lysosomal fraction with green mitochondria fluorescence, resulting in a decrease of the yellow-orange signal in the overlaid areas of the MTG and LTR, when compared to control pcDNA cells (Figure 6A). However, parkin overexpression markedly restored the colocalization of red and green fluorescence in cells treated with α-Syn. Further analysis showed that, in the presence of α-Syn, the mitophagy index in pcDNA cells was significantly decreased (*p* < 0.05), with a Pearson’s correlation coefficient of about 0.42 ± SD, whereas parkin overexpression induced an increase in this value, suggesting that the process of mitophagy was restored (Figure 6B). At the same time, no significant change in the fluorescence intensity of LysoTracker Red, a marker of lysosome content, was detected in pcDNA and pcDNA-Parkin cells treated with exogenous α-Syn (Supplementary Figure 3). To assess possible changes in the autophagic flux, in the following studies, we added the lysosomotropic agent chloroqine (CQ, 40 μM), together with α-Syn, for 24 h. This treatment paradigm was previously shown to induce a significant increase in LTR staining, due to a compensatory lysosomal biogenic response, along with the lysosomal functional impairment associated with a decrease in lysosomal enzymes’ activity (Lu et al., 2017). Indeed, CQ treatment induced an increase in LTR signal (Supplementary Figure 3), followed by an elevation of LTR and MTR colocalization (Figure 6C). Under those experimental conditions, the colocalization of the lysosomal fraction with green mitochondrial fluorescence in pcDNA cells treated with α-Syn was again significantly decreased, whereas parkin overexpression restored the α-Syn-induced deregulation of mitophagy (Figures 6A,C). Again, no significant change in the fluorescence intensity of LysoTracker Red, a marker of lysosome content, was detected in pcDNA and pcDNA-Parkin cells treated with exogenous α-Syn (Supplementary Figure 3). Subsequently, we analyzed the involvement of α-Syn in total autophagic flux, by detection of microtubule-associated protein 1A light chain 3 I (LC3 I) conversion to insoluble LC3 II form in the presence and absence of CQ. The immunoblot analysis showed the negligible effect of α-Syn treatment of pcDNA cells and parkin overexpression on autophagic flux (Figure 6D).

**Figure 7:**
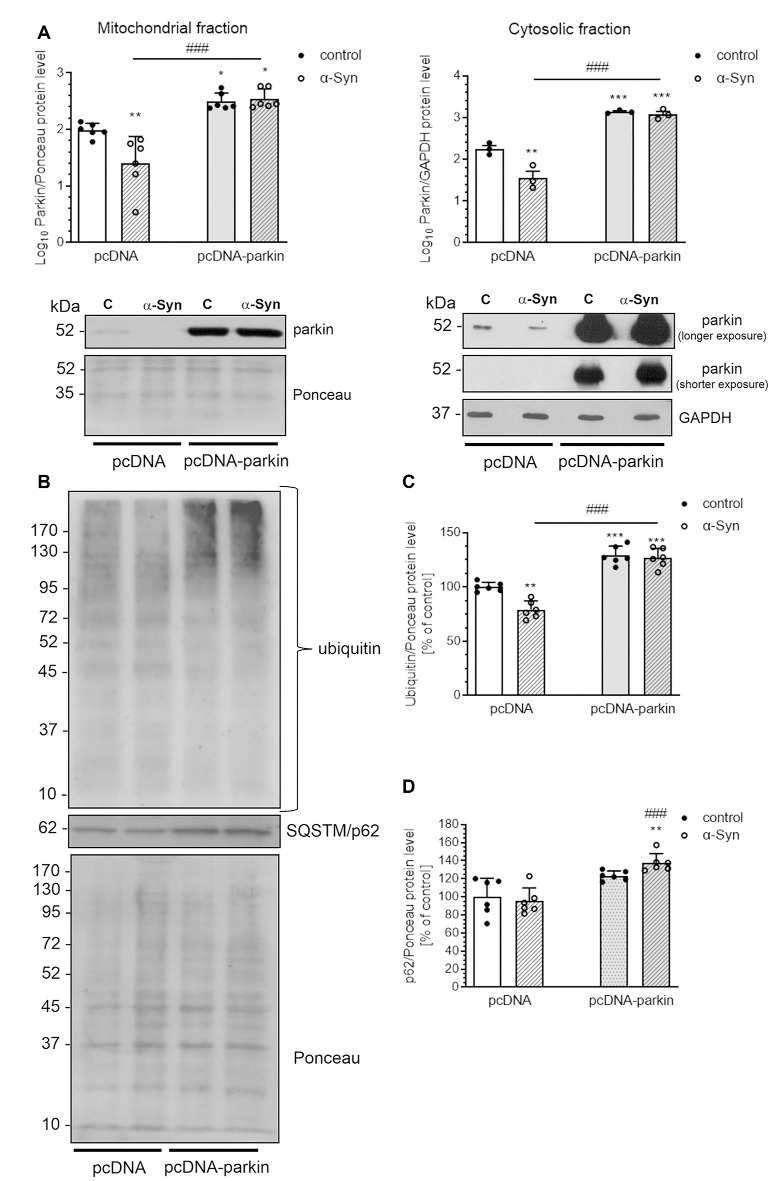
α-Syn treatment decreases the mitochondrial level of parkin in PC12 cells. **(A)** The immunoreactivity of parkin in mitochondrial (normalized relative to Ponceau-S) and cytosolic (normalized to GAPDH) extracts from pcDNA and pcDNA-Parkin PC12 cells treated with α-Syn for 24 h at a concentration of 5 μM. Data were log10 transformed and represent the mean value ± SD (mitochondria—*n* = 6, cytosol—*n* = 3) *p < 0.05, **p < 0.01, ***p < 0.001 compared to pcDNA control cells; ^###^*p* < 0.001 compared to pcDNA cells treated with α-Syn, using two-way ANOVA followed by Bonferroni *post hoc* test. **(B)** Representative photograph of Western blot analysis of ubiquitin and p62 in mitochondrial extracts from pcDNA and pcDNA-Parkin PC12 cells treated with α-Syn for 24 h at a concentration of 5 μM. **(C)** Immunoreactivity of ubiquitin normalized to Ponceau-S. Data were normalized to the untreated control group (=100%) and represent the mean value ± SD for six independent experiments (*n* = 6). **p < 0.01, ***p < 0.001 compared to pcDNA control cells; ^###^*p* < 0.001 compared to pcDNA cells treated with α-Syn, using two-way ANOVA followed by Bonferroni *post hoc* test. **(D)** Immunoreactivity of p62 normalized to Ponceau-S. Data were normalized to the untreated control group (= 100%) and represent the mean value ± SD for six independent experiments (*n* = 6). **p < 0.01 compared to pcDNA control cells; ^###^*p* < 0.001 compared to pcDNA cells treated with α-Syn, using two-way ANOVA followed by Bonferroni *post hoc* test.

As parkin translocation to mitochondria was proven to be a critical step in the process of mitophagy, we subsequently assessed the immunoreactivity of this protein in the mitochondria and cytosol of pcDNA cells treated with α-Syn and observed a substantial decline in parkin levels in both fractions (Figure 7A). On the other hand, parkin overexpression markedly elevated the level of this protein in cytosol and mitochondria of pcDNA-Parkin control and α-Syn-treated cells (Figure 7A), We also observed that α-Syn-induced decrease in mitochondrial parkin level was followed by a reduction in ubiquitination of mitochondrial proteins, which was robustly increased in pcDNA-Parkin control and α-Syn-treated cells (Figures 7B,C). Ubiquitination is a mechanism for priming the mitochondria for autophagic clearance by attracting the ubiquitin- and LC3-binding adaptor protein SQSTM/p62 (hereafter referred to as p62) to mitochondria, which is a crucial step in mitophagy (Geisler et al., 2010). We observed that, although α-Syn did not significantly change the p62 level in the mitochondria in pcDNA cells, parkin overexpression induced a significant increase of mitochondrial p62 in α-Syn-treated PC12 cells (Figures 7B,D).

**Figure 8:**
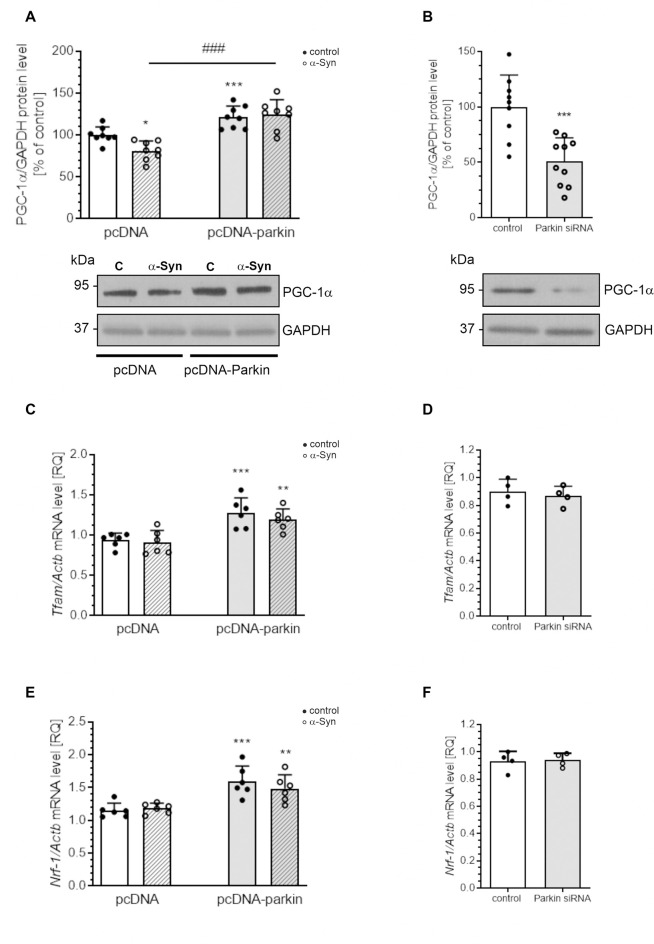
Parkin deregulation induced by α-Syn treatment decreases PGC1α expression in PC12 cells. **(A)** PGC1α immunoreactivity normalized to GAPDH in pcDNA and pcDNA-Parkin PC12 cells treated with α-Syn for 24 h at a concentration of 5 μM. Data were normalized to the untreated control group (=100%) and represent the mean value ± SD for eight independent experiments (*n* = 8). *p < 0.05, ***p < 0.001 compared to pcDNA control cells; ^###^*p* < 0.001 compared to pcDNA cells treated with α-Syn, using two-way ANOVA followed by Bonferroni *post hoc* test. **(B)** PGC1α immunoreactivity normalized to GAPDH in Parkin knock-down PC12 cells. Data were normalized to the untreated control group (=100%) and represent the mean value ± SD for 10 independent experiments (*n* = 10). ***p < 0.001 compared to corresponding control siRNA, using Student’s *t*-test. **(C)** The mRNA level of *Tfam* in pcDNA and pcDNA-Parkin PC12 cells treated with α-Syn for 24 h at a concentration of 5 μM. The mRNA level was measured by real-time PCR and normalized to *Actb* (β-actin). Data represent the mean value ± SD for six independent experiments (*n* = 6). **p < 0.01, ***p < 0.001 compared to pcDNA control cells using two-way ANOVA followed by Bonferroni *post hoc* test. **(D)** The mRNA level of *Tfam* in Parkin knock-down PC12 cells. The mRNA level was measured by real-time PCR and normalized to *Actb* (β-actin). Data represent the mean value ± SD for four independent experiments (*n* = 4). **(E)** The mRNA level of *Nrf-1* in pcDNA and pcDNA-Parkin PC12 cells treated with α-Syn for 24 h at a concentration of 5 μM. The mRNA level was measured by real-time PCR and normalized to *Actb* (β-actin). Data represent the mean value ± SD for six independent experiments (*n* = 6). **p < 0.01, ***p < 0.001 compared to pcDNA control cells using two-way ANOVA followed by Bonferroni *post hoc* test. **(F)** The mRNA level of *Nrf-1* in Parkin knock-down PC12 cells. The mRNA level was measured by real-time PCR and normalized to *Actb* (β-actin). Data represent the mean value ± SD for four independent experiments (*n* = 4).

**Figure 9:**
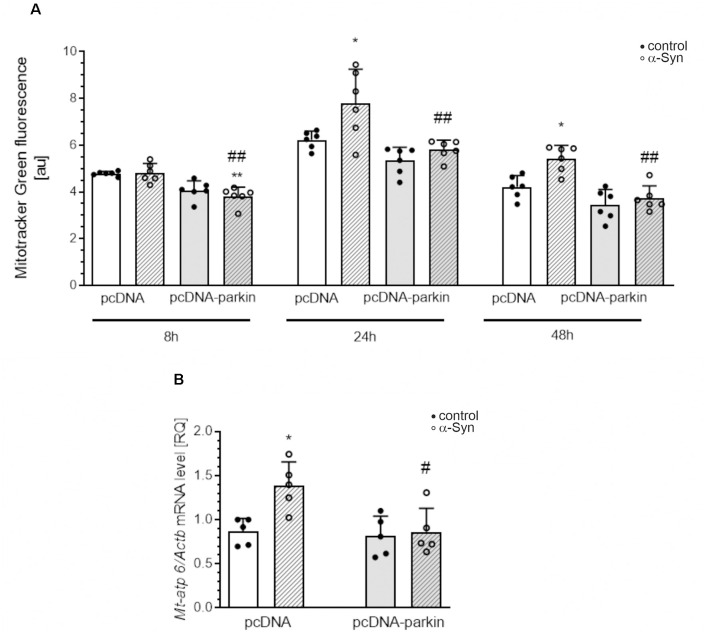
Parkin deregulation induced by α-Syn treatment increases mitochondrial mass and mtDNA in PC12 cells. **(A)** Flow cytometry analysis and quantification of mitochondrial mass in pcDNA and pcDNA-Parkin PC12 cells treated with α-Syn for 8, 24, and 48 h at a concentration of 5 μM with mitotracker green (MTG). Data represent the mean value ± SD. for six independent experiments (*n* = 6). *p < 0.05, **p < 0.01 compared to pcDNA control cells; ^##^*p* < 0.01 compared to pcDNA cells treated with α-Syn, using two-way ANOVA followed by Bonferroni *post hoc* test. **(B)** mtDNA copy number in pcDNA and pcDNA-Parkin PC12 cells treated with α-Syn for 24 h at a concentration of 5 μM quantified by real-time PCR measurement of mitochondrial DNA encoded *Mt-atp6* relative to the nuclear genome (*Gapdh* gene). Data represent the mean value ± SD for five independent experiments (*n* = 5). *p < 0.05 compared to pcDNA control cells; ^#^*p* < 0.05 compared to pcDNA cells treated with α-Syn, using two-way ANOVA followed by Bonferroni *post hoc* test.

Parkin was shown not only to trigger tagging and clearance of damaged mitochondria through mitophagy but also to interface with mitochondrial biogenesis. Therefore, in the next studies, we examined the markers of mitochondrial biogenesis: peroxisome proliferator-activated receptor gamma-coactivator 1-α (PGC-1α); as well as mitochondrial transcription factor A (TFAM) and nuclear respiratory factor-1 (NRF-1; Figure 8). PGC-1α stimulates the expression of many proteins implicated in the regulation of cell energy metabolism and is a strong stimulator of mitochondrial biogenesis. In our study, treatment with exogenous α-Syn induced a decrease in PGC-1α level in pcDNA cells (Figure 8A). This phenomenon seems to be related to parkin protein level in PC12 cells, as parkin overexpression induced elevation of the immunoreactivity of PGC-1α (Figure 8A), whereas parkin silencing resulted in a considerable decrease in this protein level (Figure 8B). Interestingly, the expression of TFAM, the key enhancer protein regulating the expression of mtDNA genes, and NRF-1, responsible for regulating the expression of proteins encoded by both mitochondrial and genomic DNA, was significantly elevated in cells overexpressing parkin (Figures 8C,E); parkin silencing did not change the mRNA level for either of these factors (Figures 8D,F). Finally, we studied the consequences of α-Syn treatment for mitochondrial mass over the time course. Using MitoTracker-Green, we found that, upon an α-Syn treatment, a significant increase in mitochondria content is already detectable after 24 h and lasts up to 48 h in pcDNA cells, whereas in cells overexpressing parkin, we observed that α-Syn-treatment induced a significant decrease in the MitoTracker-Green fluorescence after 8 h, but in the following time points the effect of α-Syn on mitochondria mass in this cell line was negligible (Figure 9A). To confirm that mitochondrial number was increased in PC12 cells treated with exogenous α-Syn, the mitochondrial DNA (mtDNA) copy number was assessed by measuring the expression of mitochondrially encoded ATP synthase membrane subunit 6’ (MT-ATP6) with reference to GAPDH (encoded by genomic DNA), using real-time quantitative PCR (Figure 9B). We observed that α-Syn treatment in pc-DNA cells resulted in a ~60% increase in mitochondrial DNA copy number as assessed by MT-ATP6 level (Figure 9B). Parkin overexpression significantly reverses the change in MT-ATP6 level induced by α-Syn treatment (Figure 9B). Together, those data suggest that, upon the α-Syn treatment, the removal of damaged mitochondria is impaired in parkin deficient cells.

## Discussion

A plethora of studies have demonstrated that deposition of α-Syn in Lewy bodies, parkin impairment, and mitochondria dysfunction are key features of PD pathology (Jesko et al., 2019). The underlying pathogenesis, however, is largely unclear and causal treatment strategies are still missing. This study is the first to show that exogenous α-Syn leads to disturbances in parkin-dependent mechanisms of mitophagy, resulting in the accumulation of defective mitochondria and that the overexpression of parkin ameliorates the mitochondrial dysfunction evoked by α-Syn oligomers.

Many previous reports suggested mitochondria as the major target of α-Syn-evoked toxicity in neuronal cells (Loeb et al., 2010; Wilkaniec et al., 2013; Ganjam et al., 2019). It was shown that cells overexpressing wt or mutated α-Syn displayed several mitochondrial defects, such as loss of mitochondrial membrane potential and mitochondrial fragmentation followed by elevation of oxidative stress (Pozo Devoto et al., 2017; Ganjam et al., 2019). Mitochondrial depolarization and decreased ATP levels were also observed upon treatment with exogenous wt and mutant α-Syn (Banerjee et al., 2010). Accordingly, our results show that extracellular α-Syn induces a substantial decline in mitochondrial membrane potential and in ATP synthesis. Also, our previous studies indicated that exogenous oligomers of α-Syn induce a significant decrease in the level of parkin (Wilkaniec et al., 2019), which is well known to promote mitochondrial homeostasis. A plethora of studies demonstrated that parkin mutations or gene silencing relate to mitochondrial dysfunction and a decline in mitochondrial OXPHOS activity (Palacino et al., 2004; Thomas et al., 2007; Mortiboys et al., 2008), but *per se*, they are insufficient to induce significant neurodegeneration within SNpc (Goldberg et al., 2003). Here, we showed that either α-Syn treatment that induces a decrease in parkin protein level or the siRNA-mediated parkin gene silencing causes the mitochondrial dysfunction. However, the toxic effect of α-Syn on mitochondria was significantly higher, than those induced by the parkin silencing. These observations suggest that a decrease in parkin level is not the only mechanism responsible for the α-Syn-dependent impairment of mitochondrial function and the involvement of other mechanisms cannot be excluded. Previously, the direct, concentration-dependent translocation of either wt or A30P and A53T mutant α-Syn to mitochondria was reported both *in vitro* and in various PD animal models (Parihar et al., 2009; Subramaniam et al., 2014), as well as in substantia nigra of PD patients (Devi et al., 2008). Interestingly, it was demonstrated that exogenously administered aggregated α-Syn species (oligomers and fibrils) are efficiently internalized by the recipient cells (Hoffmann et al., 2019) and in primary neurons, they preferentially bind to mitochondria and induce significant defects in cellular respiration (Wang et al., 2019). Intramitochondrial α-Syn was shown to interact with mitochondria-associated endoplasmic reticulum membranes (MAMs; Guardia-Laguarta et al., 2014), as well as complex I, leading to disturbances in its function and elevation of free radical production (Devi et al., 2008; Liu et al., 2009). Apart from its direct association with mitochondria, α-Syn may also indirectly affect their function. It was previously demonstrated that monomeric species of α-Syn, that are internalized less efficiently than oligomers (Hoffmann et al., 2019), can preferentially bind to and activate neuronal purinergic P2X7 receptor (Wilkaniec et al., 2017), which leads to a decrease in mitochondria membrane potential as well as elevation of mitochondrial ROS production (Wilkaniec et al., 2020). Also, oligomeric species of α-Syn were shown to induce selective oxidation and nitrosylation of mitochondrial proteins, triggering mitochondrial swelling and neuronal cell death (Tapias et al., 2017; Ludtmann et al., 2018). However, since the α-Syn-evoked decrease in mitochondrial membrane potential and cellular ATP levels in neuronal cells was significantly attenuated by parkin overexpression, probably, α-Syn-evoked decrease in parkin level might further aggravate the toxic effect of this protein on mitochondria. We also observed that α-Syn increased the level of mitochondrial superoxide, as well as induced changes in mitochondrial redox potential in a manner greatly dependent on parkin level. This corresponds with the previous studies showing that α-Syn causes a significant reduction in the expression of proteins regulating mitochondrial antioxidative defense that leads to an increase in protein and lipid peroxidation (Palacino et al., 2004). Interestingly, we observed that in cells with parkin overexpression, the mtROS levels and mitochondrial redox state are markedly decreased when compared with control pcDNA cells. This may be attributed to the unique function of parkin as a direct mediator of antioxidative reactions, including free radical reduction and glutathione regeneration (El Kodsi et al., 2020), but it may also depend largely on the superoxide-driven stimulation of parkin-mediated mitophagy (Xiao et al., 2017a). Taken together, our studies show that the overexpression of parkin ameliorates the deleterious effects of oligomeric α-Syn on mitochondria, which support a protective role for parkin in neurodegeneration.

One possible mechanism through which parkin overexpression prevents mitochondrial dysfunction induced by α-Syn is the regulation of mitochondrial fusion and fission processes. Previous studies indicated aberrant mitochondrial fragmentation as an important component of PD pathology (Lee et al., 2012), implicating the role of a pathological pool of α-Syn on the fragmentation of the mitochondrial network (O’Donnell et al., 2014; Burté et al., 2015; Pozo Devoto et al., 2017). For example, in A53T-overexpressing neuroblastoma cells, the altered mitochondrial morphology and the increased translocation of fission regulator—Drp1 to mitochondria was demonstrated (Gui et al., 2012). In agreement, our data showed that extracellular oligomeric α-Syn activates mitochondrial fission and suggest that this effect of α-Syn is largely dependent on the translocation of fission-regulating Drp1 protein to mitochondria. Moreover, our results demonstrated that exogenous α-Syn significantly decreased the level of Mfn2. Those data are in agreement with the earlier report showing that, in fibroblasts from Drp1 knockout mice, α-Syn can still induce mitochondrial fragmentation (Nakamura et al., 2011). Interestingly, in the present study, the increase in parkin level partly prevented mitochondrial fragmentation induced by α-Syn, but parkin overexpression did not influence the α-Syn-evoked Drp1 translocation to mitochondria. In agreement with this observation, previous studies showed that Drp1 was not the direct substrate for parkin-mediated ubiquitination (Glauser et al., 2011), and changes in parkin levels did not influence the translocation of Drp1 (Poole et al., 2010). Moreover, we observed that parkin overexpression induced downregulation of Mfn2 *per se*. These observations partly correspond with previous studies showing that parkin can ubiquitinate Mfn1 and Mfn2, leading to their degradation, but this process was suggested to be secondary to depolarization-induced fission and its major role was preventing or delaying re-fusion of defective mitochondria with functional organelles (Tanaka et al., 2010; Gegg and Schapira, 2011). It seems that parkin is not essential to regulating mitochondria dynamics, as the expression of mitochondria-shaping proteins is also controlled by the other ubiquitin ligases, such as Huwe 1 (Leboucher et al., 2012), mitochondrial ubiquitin ligase MITOL/MARCH V (Yonashiro et al., 2006), MUL1 (Braschi et al., 2009), and Bcl-2 proteins (Rolland and Conradt, 2010). In agreement with those data, we observed that silencing parkin in PC12 cells neither influences the level of fusion/ fission proteins nor prevents the decrease in Mfn2 level induced by α-Syn. Nevertheless, it remains unclear how parkin prevents mitochondrial fragmentation induced by α-Syn. Recent data suggest that α-Syn affects mitochondrial size by working independently from core fusion/fission proteins (Nakamura et al., 2011; Guardia-Laguarta et al., 2014; Liu et al., 2015) because it was able to inhibit mitochondrial elongation in cells overexpressing Mfn1, Mfn2, and Opa1 (Kamp et al., 2010). Moreover, α-Syn knockdown (Kamp et al., 2010) or disruption of its N-terminal fragment, which is crucial for membrane binding, resulted in the elevation of mitochondrial assembly without inducing changes in expression of fusion-fission proteins (Pozo Devoto et al., 2017). It was also demonstrated that α-Syn translocation to mitochondria induces their fragmentation (Pozo Devoto et al., 2017), probably through direct interaction with the outer mitochondrial membrane, leading to mechanical blockade of mitochondrial fusion (Kamp et al., 2010). Therefore, we may speculate that parkin somehow prevents the direct association of α-Syn with mitochondria. As α-Syn decreased parkin levels, due to its S-nitrosylation (Wilkaniec et al., 2019), it is therefore possible that downregulating parkin may indirectly intensify the adverse effects of α-Syn oligomers on mitochondrial dynamics.

Taking into consideration that regulation of the mitochondrial life cycle is one of the most important molecular functions of parkin (Ryan et al., 2015; Hammerling et al., 2017), and that the accumulation of dysfunctional mitochondria is directly responsible for inducing neuronal cells death in PD patients with parkin gene mutations (Narendra et al., 2008; Burman et al., 2012; de Vries and Przedborski, 2013), α-Syn-induced parkin downregulation may be the major cause of mitophagy disruption and the accumulation of malfunctioning mitochondria. However, a consensus concerning the effect of intracellular α-Syn overload on the process of mitochondria degradation has not been reached. The increased mitophagy associated with energy deficits and neuronal degeneration was observed in primary cortical neurons and in transgenic animals overexpressing mutant A53T α-Syn (Chinta et al., 2010; Choubey et al., 2011). By contrast, some studies of transgenic animals, as well as of the brains of PD patients, showed a greater accumulation of mitochondria in degenerated neurons (Chen et al., 2018; Shaltouki et al., 2018). In agreement with those studies, our results demonstrate that exogenous α-Syn oligomers decrease mitophagy in dopaminergic cells by disrupting the interaction of mitochondria with lysosomes, resulting in the accumulation of dysfunctional organelles. Earlier evidence obtained from PD patients and PD models demonstrated disturbances in lysosome function, as well as an excessive accumulation of autophagic vacuoles (Chu et al., 2009; Prigione et al., 2010). As overexpression of wt α-Syn was demonstrated to inhibit autophagosome formation, diminish LC3-II levels and induce accumulation of autophagy substrates (Winslow et al., 2010), the observed abnormalities in the elimination of damaged mitochondria may be associated with macroautophagy impairment. However, we observed that, when exogenously administered, wt α-Syn has a negligible effect on the autophagosomes’ formation. Also, the reports on transgenic animal models showed that the elevation of lysosomal indicators in aged DA neurons is attributed to A53T mutation (Lin et al., 2012), while others have suggested that only aggregated forms of α-Syn can efficiently disrupt autophagic activity in neurons (Tanik et al., 2013). Taken together, those studies indicated that the negative impact of α-Syn on macroautophagy is mainly attributable to its intracellular activity that is largely dependent on the point-and copy-number mutations or aggregation state of the protein. In agreement with those data, our study indicated that impairment of mitophagy induced by extracellular α-Syn seems to be unrelated to disturbances in autophagic flux, but it is mainly attributed to changes in parkin protein level, as in the cells overexpressing parkin, α-Syn treatment had a negligible effect on the interaction of mitochondria with lysosomes. Interestingly, in the present study, we found that exogenous α-Syn not only reduces the total level of parkin but also evokes a significant down-regulation of parkin level in the mitochondrial fraction, with a concomitant decrease of polyubiquitination of mitochondrial proteins, which is the initial step of the mitophagy process (Chan et al., 2011; Sarraf et al., 2013), leading to the recruitment of autophagic adaptor proteins (Ding et al., 2010; Wong and Holzbaur, 2014). Moreover, we showed that those α-Syn-induced changes were reversed by parkin overexpression that additionally led to elevated mitochondrial recruitment of the ubiquitin- and LC3-binding adaptor protein p62, which not only functions as a selective macroautophagy receptor but also was demonstrated to mediate aggregation of dysfunctional mitochondria into tight clusters to protect against apoptosis induced by mitochondrial depolarization (Narendra et al., 2010; Xiao et al., 2017b).

Finally, in the present study, we demonstrated that, along with impaired mitophagy, exogenous α-Syn causes disturbances in the de novo synthesis of mitochondria through inhibition of PGC-1α expression. Recent studies have suggested that PGC-1α deficiency is directly involved in PD pathogenesis, as downregulation of many genes regulated by PGC-1α was detected in PD patients and in patients with Lewy body disease (Zheng et al., 2010). Moreover, PGC-1α overexpression in midbrain primary neuronal cultures rescued cell loss caused by A53T α-Syn (Zheng et al., 2010). It was previously demonstrated that PGC-1α is an indirect substrate of parkin that may regulate its level through inactivation of Parkin Interacting Substrate (PARIS), a transcriptional repressor of PGC-1α (Shin et al., 2011). Under physiological conditions, parkin ubiquitinated PARIS, leading to its proteasomal degradation, thereby abolishing its inhibitory effect on PGC-1 α and enabling mitochondrial biogenesis. Our study showed that parkin silencing leads to a significant downregulation of the PGC-1α level and parkin overexpression rescues α-Syn-mediated decrease in the expression of this transcription regulator. It is, however, possible that, by inducing a decrease in parkin level, α-Syn interferes with PARIS degradation, which in turn downregulates PGC-1α. However, some data suggest that α-Syn may have a direct effect on PGC-1α expression, because, under conditions of oxidative stress, α-Syn translocates to the nucleus and acts as a transcriptional modulator of PGC-1α (Siddiqui et al., 2012). Therefore, it can be suggested that the opposing effects of α-Syn and parkin on PGC-1α levels can be exerted by independent mechanisms, and the reduction of parkin levels due to α-Syn-dependent S-nitrosylation may negatively tip the physiological balance, leading to deregulation of mitochondrial biogenesis. PGC-1α is also indirectly involved in regulating the transcription of mtDNA genes *via* mitochondrial transcription factor TFAM, which is co-activated by NRF-1 (Taherzadeh-Fard et al., 2011). Interestingly, our study demonstrated that, though α-Syn treatment leads to a decrease in PGC-1α level, it has a negligible effect on NRF-1 and TFAM expression. One possible explanation of this phenomenon is that the expression of NRF-1 and its downstream target, TFAM, may be regulated independently of PGC-1 α levels. It was previously shown that NFκB and CREB activation co-regulate the NRF-1 promoter, leading to the downstream expression of TFAM (Suliman et al., 2010). Another possibility is that PGC-1 α is regulated with different kinetics compared to TFAM and NRF-1, or that these factors may be more stable than PGC-1α; thus, in our experimental paradigm, the downregulation of PGC-1α does not necessarily cause immediate downregulation of the other two transcription factors, which may occur at later time points. Interestingly, parkin overexpression resulted in a significant elevation of mRNA level for both NRF-1 and TFAM that may further stimulate mitochondrial biogenesis. Similarly, other studies also demonstrated that parkin overexpression enhances transcription and replication of mitochondrial DNA that was associated with enhanced TFAM activity (Kuroda et al., 2006). Those observations are in line with Dawson’s hypothesis that an increase in parkin level above control values may activate many signaling pathways that are not regulated by endogenous levels of this protein (Dawson and Dawson, 2010). Nevertheless, our results indicate the significant role of parkin downregulation in disturbances of mitochondrial biogenesis, which may affect the level of functional mitochondria. We suggest that loss of parkin function as a result of α-Syn treatment evoked an overall collapse in mitochondrial homeostasis: on the one hand, it induced disturbances in the degradation of defective mitochondria, and on the other, it interfered with processes related to mitochondria biogenesis, which ultimately led to the accumulation of abnormal mitochondria. Those observations are in line with previous studies showing that loss of parkin function caused an accumulation of mitochondria with a simultaneous decrease in ATP synthesis (Matsuda et al., 2010; Vives-Bauza et al., 2010). Moreover, the deposition of dysfunctional mitochondria may have an impact on dopaminergic cell death through elevation of free radical generation and increased liberation of mitochondria-derived pro-apoptotic factors (Vila and Przedborski, 2003; Perier et al., 2007). In turn, under the conditions of cellular stress evoked by α-Syn treatment, restoring the level of parkin resulted in increased degradation of damaged mitochondria and stimulated the synthesis of new functional organelles.

In sum, we demonstrated that parkin loss of function induced by α-Syn oligomers was responsible for disrupting the balance between the clearance of defective mitochondria and the generation of new functional organelles, thereby likely amplifying α-Syn toxicity through the accumulation of dysfunctional mitochondria. By providing the first compelling evidence for the direct association of α-Syn-mediated parkin depletion to impaired mitochondrial function, this study extends previous findings and provides a foundation for future studies on PD pathomechanisms.

## Data Availability Statement

The raw data supporting the conclusions of this article will be made available by the authors, without undue reservation.

## Author Contributions

AW and AA: conceptualization, project administration, and funding acquisition. AW, AL, LB, EM, MC, HJ, and CC: methodology. AA and CC: validation, writing—review and editing. AW: writing—original draft preparation, AW, AL, and MC: visualization. AA: supervision. All authors have read and agreed to the published version of the manuscript.

## Conflict of Interest

The authors declare that the research was conducted in the absence of any commercial or financial relationships that could be construed as a potential conflict of interest.

## Abbreviations

α-syn: α-synuclein
Actb: Actin
ATP: Adenosine triphosphate
CNS: central nervous system
DA: dopamine
Drp-1: Dynamin related protein 1
GAPDH: Glyceraldehyde 3-phosphate dehydrogenase
KO: knockout
LB: Lewy bodies
LC3: microtubule-associated protein 1A light chain 3
LTR: Lysotracker Red
MAMs: mitochondria-associated endoplasmic reticulum membranes
Mfn1: Mitofusin 1
Mfn2: Mitofusin 2
MMP: mitochondrial membrane potential
MT-ATP6: mitochondrially encoded ATP synthase membrane subunit 6′
mtDNA: mitochondrial DNA
MTG: Mitotracker Green
mtROS: mitochondrial reactive oxygen species
NGF: nerve growth factor
NRF-1: nuclear respiratory factor-1
OMM: outer mitochondrial membrane
Opa1: Optic atrophy 1
PARIS: Parkin Interacting Substrate
PC12: rat pheochromocytoma cell line
pcDNA: mock PC12 cells
pcDNA-Parkin: parkin overexpressed PC12 cells
PD: Parkinson’s disease
PGC-1α: peroxisome proliferator-activated receptor gamma-coactivator 1-alpha
PINK-1: PTEN-induced kinase 1
RT-PCR: real-time quantitative PCR
siRNA-Parkin: naïve PC12 cells with silenced endogenous parkin
SNpc: substantia nigra pars compacta
SOD1: cytoplasmic superoxide dismutase 1
SOD2: mitochondrial superoxide dismutase 2
SQSTM/p62: ubiquitin- and LC3-binding adaptor protein
TFAM: transcription factor A, mitochondrial
TOM20: Mitochondrial import receptor subunit 20
UPS: ubiquitin-proteasome system.
