# Supplementary material for: Exogenous Alpha-Synuclein Evoked Parkin Downregulation Promotes Mitochondrial Dysfunction in Neuronal Cells. Implications for Parkinson’s Disease Pathology
Source: Front Aging Neurosci. 2021 Feb 24;13:591475. doi: 10.3389/fnagi.2021.591475 (PMC7943853; doi:10.3389/fnagi.2021.591475)
Supplement: Supplementary file 1 [file Table_1.DOCX]

Supplementary Material


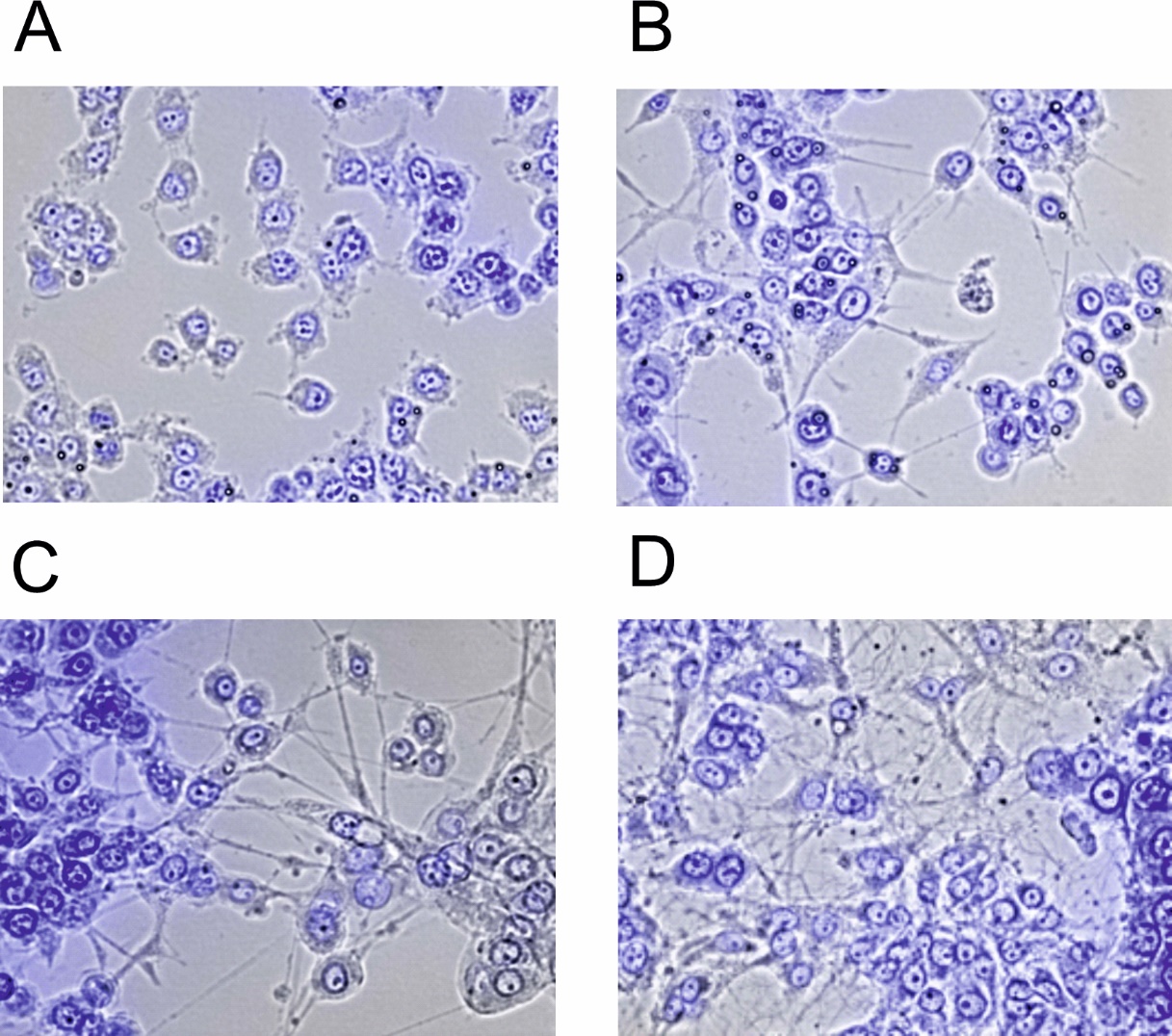


**Supplementary Figure 1.** **NGF-differentiated PC12 cells.**

PC12 cells were treated with NGF, 50 ng/ml, for 96h in low-serum medium (DMEM supplemented with 2% FBS, 1% penicillin/streptomycin, and 1% L-glutamine). NGF induced phenotypic changes characterized by neurite outgrowth visualized in Phase-contrast. **A)** Control culture; **B-D)** correspondingly on the 24, 48 and 96 hours after NGF addition.


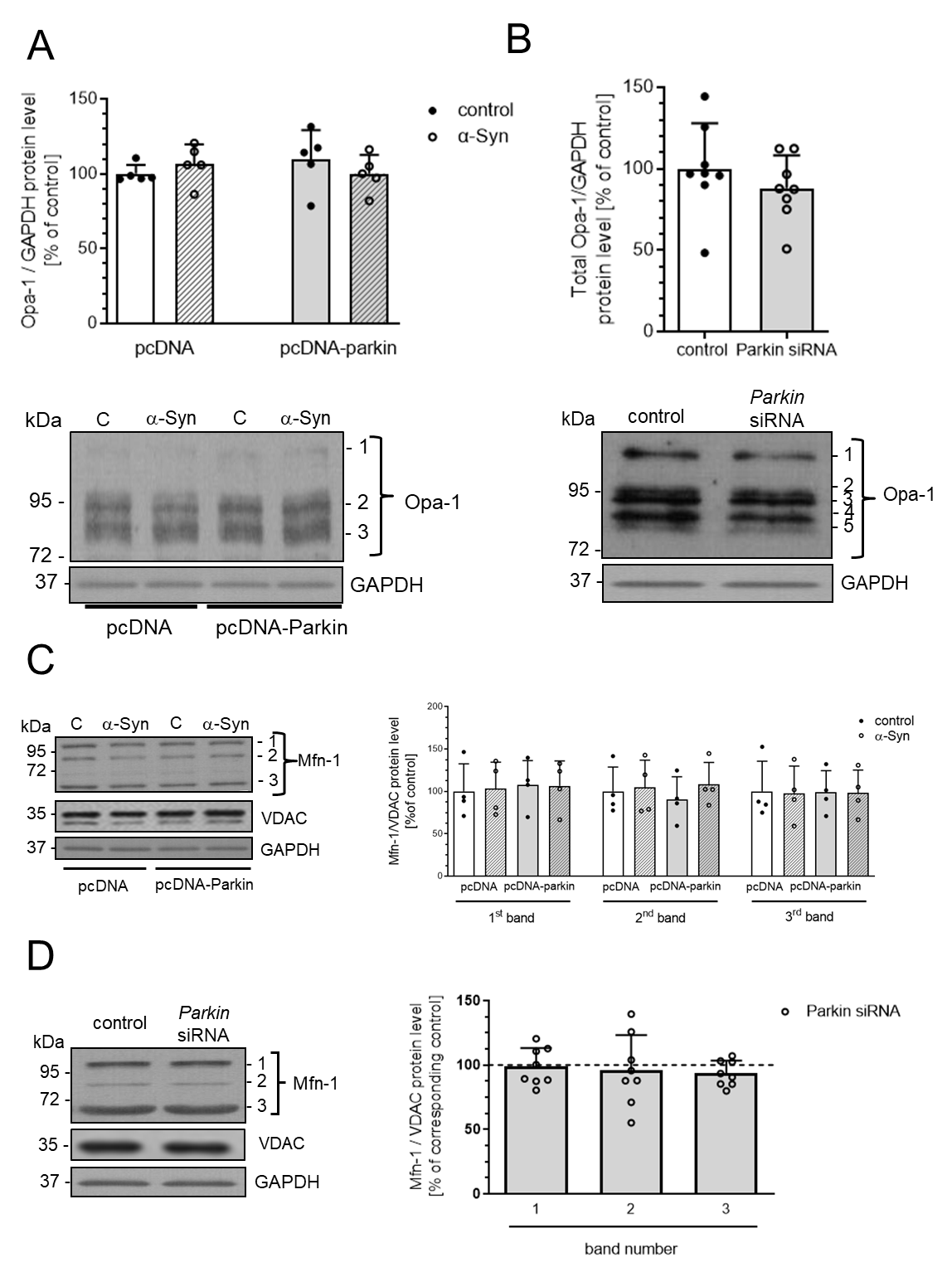


**Supplementary Figure 2.** **α-Syn treatment or parkin modifications do not change the protein level of Opa-1 and Mfn-1.**

Opa-1 immunoreactivity normalized to GAPDH in **A)** pcDNA and **B)** pcDNA-Parkin PC12 cells treated with α-Syn for 24 h at a concentration of 5 μM, or in Parkin knock-down PC12 cells. Data were normalized to the corresponding untreated control group (= 100%) and represent the mean value ± SD. (A - n=5, B - n=8). **C)** Mfn-1 immunoreactivity normalized to VDAC in pcDNA and pcDNA-Parkin PC12 cells treated with α-Syn for 24 h at a concentration of 5 μM, or **D)** in Parkin knock-down PC12 cells. Data were normalized to the corresponding untreated control group (=100%) and represent the mean value ± SD. (C - n=4, B - n=8).


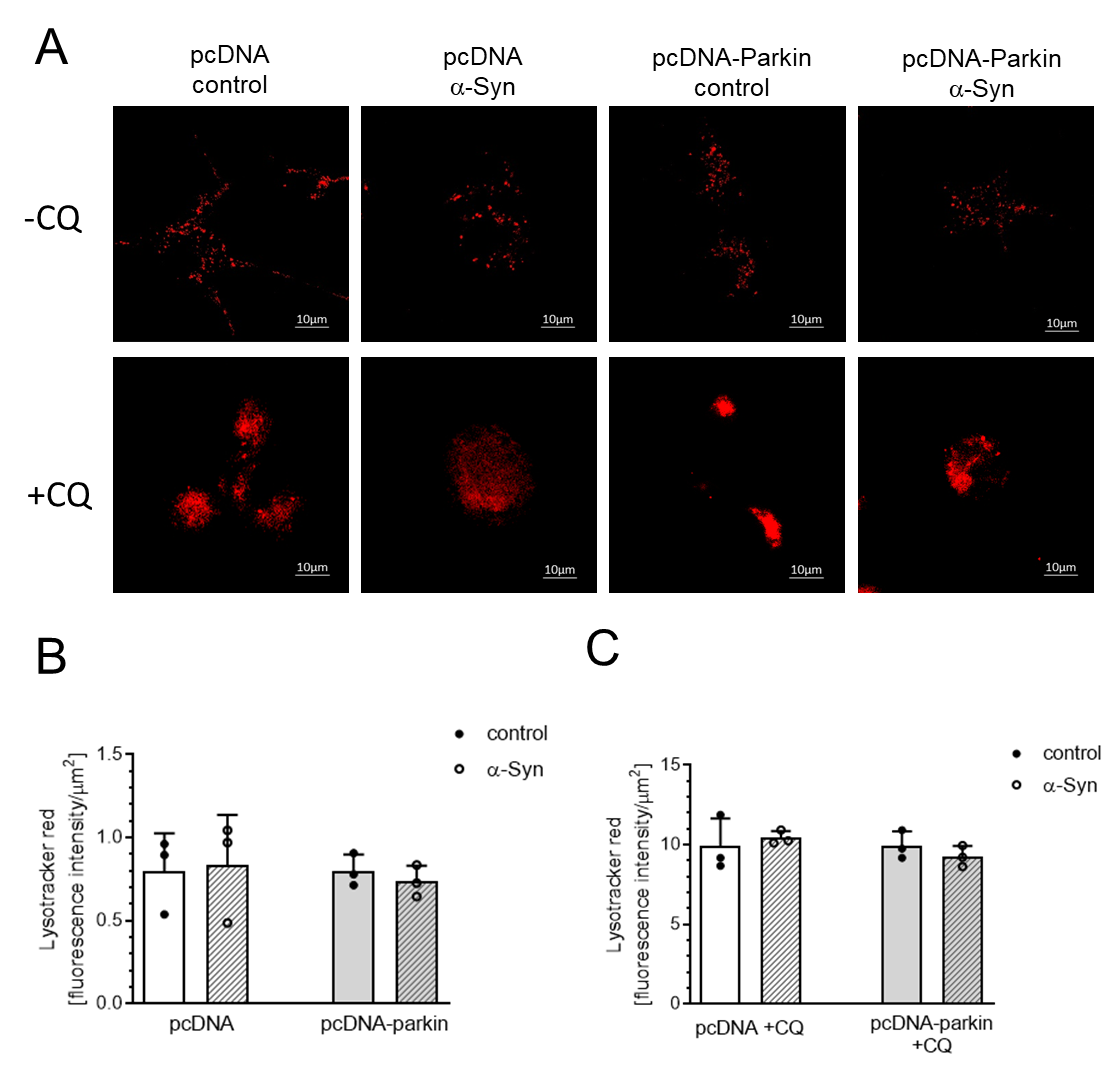
**Supplementary Figure 3.** **LysoTracker Red staining in cells treated with exogenous α-Syn oligomers.**

**A)** Single confocal captions showing LTR staining in pcDNA and pcDNA-Parkin PC12 treated for 24 h with α-Syn at a concentration of 5 μM in the absence (top panel)/ presence (bottom panel) of 40 μM chloroquine (CQ). Scale bar: 10 μm. The presented cells are representative of most of the analyzed cells. **B)** Relative value analysis of average fluorescence intensity of LysoTracker Red per cell area in PC12 cells treated for 24 h with α-Syn. Data were derived from 3 independent experiments with 8 fields per experiment. Each value is expressed as mean ± SD. C) Relative value analysis of average fluorescence intensity of LysoTracker Red per cell area in PC12 cells treated for 24 h with α-Syn in the presence of 40 μM CQ. Data were derived from 3 independent experiments with 8 fields per experiment. Each value is expressed as mean ± SD.
